# Pexophagy and Oxidative Stress: Focus on Peroxisomal Proteins and Reactive Oxygen Species (ROS) Signaling Pathways

**DOI:** 10.3390/antiox14020126

**Published:** 2025-01-23

**Authors:** Xiaofan Wei, Laxman Manandhar, Hyunsoo Kim, Arun Chhetri, Jaetaek Hwang, Gyuho Jang, Channy Park, Raekil Park

**Affiliations:** Department of Biomedical Science and Engineering, Gwangju Institute of Science and Technology, Gwangju 61005, Republic of Korea; weixiaofan1@gm.gist.ac.kr (X.W.); laxmanandhar@gm.gist.ac.kr (L.M.); ruyhyunsookim@gm.gist.ac.kr (H.K.); chhetri5@gm.gist.ac.kr (A.C.); isoul9103@gm.gist.ac.kr (J.H.); gyuho9008@gm.gist.ac.kr (G.J.); channypark@gist.ac.kr (C.P.)

**Keywords:** peroxisome, pexophagy, autophagy, ROS

## Abstract

Peroxisomes generate reactive oxygen species (ROS) and also play a role in protecting cells from the damaging effects of such radicals. Dysfunctional peroxisomes are recognized by receptors and degraded by a selective type of macroautophagy called pexophagy. Oxidative stress is one of the signals that activates pexophagy through multiple signaling pathways. Conversely, impaired pexophagy results in the accumulation of damaged peroxisomes, which in turn leads to elevated ROS levels and oxidative stress, resulting as cellular dysfunction and the progression of diseases such as neurodegeneration, cancer, and metabolic disorders. This review explores the molecular mechanisms driving pexophagy and its regulation by oxidative stress with a particular focus on ROS. This highlights the role of peroxisomal proteins and ROS-mediated signaling pathways in regulating pexophagy. In addition, emerging evidence suggests that the dysregulation of pexophagy is closely linked to neurological disorders, underscoring its potential as a therapeutic target. Understanding the intricate crosstalk between pexophagy and oxidative stress provides new insights into the maintenance of cellular homeostasis and offers promising directions for addressing neurological disorders that are tightly associated with pexophagy and oxidative stress.

## 1. Introduction

The quality of organelle and balanced oxidative stress is critical for maintaining cellular homeostasis. The clearance of damaged organelles removes potentially toxic byproducts from the cells and reuses organelle components for bioenergetics. Therefore, defects in organelle clearance can be detrimental to cell health and contribute to cancer, neurodegeneration, and inflammatory diseases [1].

Autophagy is a fundamental cellular process that occurs at the basal level and plays an important role in cellular equilibrium and removing debris from damaged organelles or cells. It can also be activated in response to cellular stresses such as starvation, hypoxia, or exposure to anticancer agents and triggers a cell survival process that supplies energy to cells during energy depletion [2]. The process of autophagy involves sequestering cytoplasmic components into double-membrane vesicles known as autophagosomes. These autophagosomes then fuse with lysosomes to degrade their contents. These degradative processes are important not only for maintaining cellular homeostasis and removing excess or damaged organelles, aggregated proteins, or pathogens from cells, but also for providing energy to support cell survival and function. Defects in autophagy have been linked to a variety of human diseases, including cancer, neurodegeneration, and inflammatory diseases [3,4]. Organelle-specific autophagy can clear mitochondria, peroxisomes, lysosomes, endoplasmic reticulum (ER), chloroplasts, and the nucleus. The selective autophagy of organelles is important for maintaining the integrity and number of organelles in the context of various environments and stresses. Organelle clearance, or selective autophagy of organelles, is different from the bulk degradation process that occurs, for example, in starvation-induced autophagy. It is a selective process that involves the specific sequestration of cellular components. Various types of organelle clearance have been identified, all of which involve initiation by a signal that triggers downstream events, leading to the recognition and marking of the target organelle for degradation. Additionally, there are also specialized molecules that mark components as cargo for degradation and autophagy-related components, promoting their sequestration and removal. Several organelles, including mitochondria, peroxisomes, lysosomes, the ER, and the nucleus, have all been identified as cargo that can be degraded by autophagy across a variety of taxa [1].

Oxidative stress results from an imbalance between the production of reactive oxygen species (ROS), including free radicals, and cellular antioxidant defense. This imbalance can lead to the damage of cellular components such as proteins, lipids, and DNA, ultimately disrupting normal cellular function. ROS, generated as byproducts of normal cellular metabolism, particularly from peroxisomes and mitochondria, can function as signaling molecules [5]. Importantly, this process is crucial for maintaining cellular health by preventing the accumulation of impaired peroxisomes, which further contributes to metabolic disturbances and the exacerbation of oxidative stress [6,7,8]. Furthermore, the only currently available study comparing the relative ROS production of different cellular sources showed that the endoplasmic reticulum (ER) and peroxisomes may have a greater capacity to generate ROS than mitochondria, at least in rat liver [9]. The peroxisomal antioxidant defense system of mammalian peroxisomes contain a variety of ROS-metabolizing enzymes, including catalase, superoxide dismutase 1, peroxyredoxin 5, glutathione S-transferase kappa, ‘microsomal’ glutathione S-transferase, and epoxide hydrolase 2. There is also evidence that these organelles utilize non-enzymatic, low-molecular-weight antioxidant compounds [10].

Peroxisomes are highly dynamic oxidative organelles with important metabolic functions in cellular lipid metabolism, including the β-oxidation of fatty acids, the synthesis of myelin sheath lipids, and the regulation of cellular redox balance. The functional loss of peroxisomes causes serious metabolic disorders in humans, such as Zellweger syndrome and X-linked adrenoleukodystrophy [11,12,13]. The idea that peroxisomes can act as endogenous stress generator stems from the discovery that the long-term administration of peroxisome proliferators to rodents induced oxidative stress in hepatocytes [14]. Peroxisomes can also protect cells from oxidative stress [15]. For example, the absence of functional peroxisomes increases apoptosis in the developing mouse cerebella [16]. In addition, mammalian cells catalyze the first two steps of plasmalogen biosynthesis exclusively by peroxisomal enzymes [17]. Cells defective in the peroxisomal steps of plasmalogen biosynthesis are shown to be significantly more sensitive than control cells to ROS generated by UV irradiation [18,19].

Peroxisomes are both sources and targets of ROS, underscoring the importance of their regulated turnover through pexophagy in maintaining cellular homeostasis. Peroxisomes are essential organelles involved in lipid metabolism and ROS detoxification. However, peroxisomal dysfunction can lead to excessive oxidative stress, thus disrupting normal cellular function [20,21]. Damaged, excessive, or dysfunctional peroxisomes are recognized to degrade by a selective autophagic degradation process known as pexophagy [22,23]. Studies have consistently shown that impaired pexophagy results in the accumulation of ROS. In contrast, ROS, as signaling molecules, play an important role in regulating pexophagy by mediating several pathways, such as inducing the ubiquitination of peroxisomal membrane proteins (PMPs). These processes underscore the intricate relationship between ROS and pexophagy, highlighting their collaborative roles in mitigating oxidative damage and maintaining cellular balance.

Peroxisomes are selectively recognized by receptors through their ubiquitin-binding domains (UBDs), and those peroxisomes interact with the LC3-interacting region (LIR) to be delivered to autophagosomes for pexophagy [24,25,26]. Two mammalian pexophagy receptors have been identified: NBR1 and p62 [27,28], which interact with ubiquitinated peroxisomal membrane proteins (PMPs) and sequester target peroxisomes into autophagosome. In addition, peroxisomes can be delivered for degradation through protein–protein interactions independently of their ubiquitin status [29,30]. Although the mechanisms and functions of pexophagy have been well studied and extensively reviewed, a critical gap still exists in our understanding of the complex regulatory networks of this process in mammalian cells.

Pexophagy specifically targets peroxisomes to degrade them, working together with peroxisome biogenesis to maintain peroxisomal homeostasis. It is tightly regulated by various signaling pathways and can be triggered beyond its basal level by several factors such as cellular stress, changes in nutrient levels and environmental stimuli. Importantly, the accumulation of dysfunctional peroxisomes can lead to metabolic disturbances and increased oxidative stress.

There is a growing number of studies suggesting that dysregulated pexophagy contributes to the development and progression of neurological diseases [31]. While ROS have been well studied in the context of neurological diseases, the role of impaired pexophagy in these conditions highlight the need for further investigation into their interplay as potential therapeutic targets [32]. In this review, we discuss the current findings of molecular mechanisms driving pexophagy, emphasizing the role of peroxisomal proteins in its regulation. In addition, we explore how ROS function as signaling molecules that trigger pexophagy, aiming to offer insights into the involute relationship between pexophagy and oxidative stress.

## 2. Pexophagy-Related Peroxisomal Proteins

**Figure 1:**
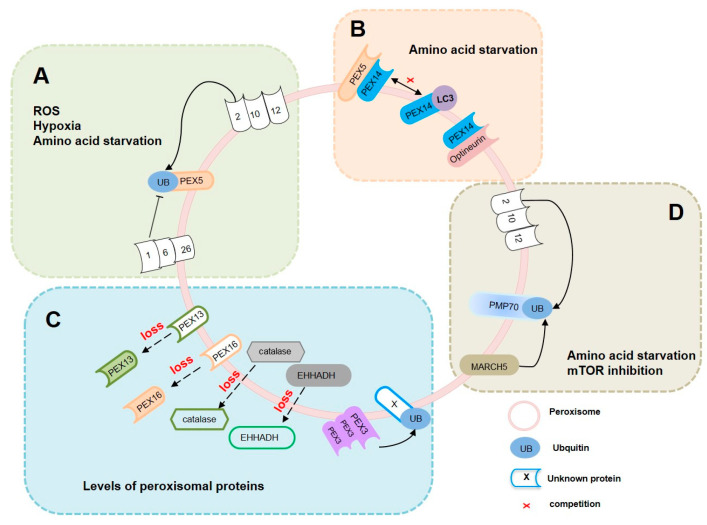
**Pexophagy-related peroxisomal proteins.** (**A**) PEX5 ubiquitination is a critical signal that promotes the initiation of pexophagy. This post-translational modification is triggered by various physiological conditions, including dysfunction of the AAA complex, amino acid starvation, and stimulation by ROS. (**B**) Under starvation conditions, PEX14 directly interacts with LC3, thereby promoting the initiation of pexophagy. Additionally, PEX14 serves as a molecular bridge linking Optineurin (OPTN) to the pexophagy pathway. (**C**) The expression levels of peroxisomal proteins significantly affect the pexophagy process. The loss of PEX13, PEX16, catalase, EHHADH, or the AAA complex leads to enhanced pexophagy. While this regulation is partially linked to ROS production and PEX5 ubiquitination, the precise mechanisms underlying these effects remain to be fully elucidated. Conversely, the overexpression of PEX3 has been shown to promote pexophagy through the ubiquitination of an unidentified protein. (**D**) PMP70, ubiquitinated by E3 ligases such as PEX2 and MARCH5, recruits pexophagy receptors, which facilitate pexophagy under diverse cellular conditions.

Peroxisomal proteins located in both the membrane and the matrix are critical for maintaining peroxisome homeostasis, which is regulated by peroxisome biogenesis and degradation [33]. Around 70–80% of peroxisomes are removed through pexophagy, a selective autophagy pathway [34]. Several proteins, such as PEX3, PEX5, and PEX16, are involved in peroxisome biogenesis. In addition, other proteins that localize or partially localize to peroxisomes and regulate pexophagy have been identified, including USP30, MARCH5, HSPA9, and p97/VCP-UBXD8 complex [35,36,37,38,39,40,41]. These proteins function differently to modulate pexophagy by regulating the ubiquitination status of peroxisomal proteins, potentially mediating the turnover of peroxisomal components, and contributing to the stress responses that influence pexophagy. Further research is necessary to fully elucidate the specific mechanisms involved in peroxisome dynamics and cellular homeostasis [35,36,37,38,39]. Defects or deficiencies in peroxisomal proteins can lead to metabolic disorders such as Zellweger syndrome, neonatal adrenoleukodystrophy, and other peroxisomal biogenesis disorders [42]. Although many of these proteins are involved in pexophagy, ongoing research continues to uncover additional factors and regulatory mechanisms that govern this selective autophagic pathway. As our understanding advances, the catalogue of peroxisomal proteins directly implicated in regulating pexophagy is expected to expand, reflecting ongoing discoveries in this field. Therefore, in this section, we will introduce the peroxisomal proteins involved in pexophagy and its associated pathways (Figure 1).

### 2.1. PEX5 Ubiquitination-Mediated Pexophagy

PEX5 plays a crucial role in peroxisomal biogenesis by facilitating the import of newly synthesized peroxisomal matrix proteins into peroxisomes. It recognizes peroxisomal targeting signal 1 (PTS1) present on the target proteins [43,44]. Once PEX5 binds to a protein carrying the PTS1 signal in the cytosol, it forms a complex with the cargo protein. This PEX5-cargo complex then docks to the peroxisomal membrane and interacts with membrane proteins such as PEX13 and PEX14 [44,45]. Upon cargo release into the peroxisome matrix, PEX5 undergoes ubiquitination and subsequent recognition by the peroxisomal membrane proteins PEX2, PEX10, and PEX12, thereby facilitating its extraction from the peroxisomal membrane and recycling for further rounds of import [46]. In addition, PEX5 plays a key role in the ubiquitination of peroxisomal membrane proteins, marking them for recognition by autophagic receptors, which then deliver damaged peroxisomes to the autophagic machinery. Several studies have demonstrated that PEX5 ubiquitination in response to several stimuli, including ROS accumulation, hypoxia, and amino acid starvation, recruits p62 and NBR1 to activate pexophagy [27,47,48]. The peroxisomal AAA ATPase complex (AAA complex) is essential for cycling PEX5 during peroxisomal matrix protein import. The disruption of the AAA complex leads to the accumulation of ubiquitinated PEX5 on the peroxisomal membrane, thereby triggering pexophagy [49]. PEX13, a component of the peroxisomal matrix import system, regulates pexophagy. The loss of PEX13 results in the accumulation of ubiquitinated PEX5 in peroxisomes, promoting the initiation of pexophagy [50]. These coordinated processes highlight the interplay between the peroxisomal protein import machinery and pexophagy regulation, which is crucial for maintaining peroxisome function and cellular homeostasis (Figure 1A).

### 2.2. PEX14-Mediated Pexophagy

PEX14 is a peroxisomal membrane protein involved in peroxisomal biogenesis. It functions as a docking receptor, ensuring that proteins are correctly delivered to their destination [51,52]. Notably, PEX5 forms a complex with peroxisomal matrix proteins in the cytosol, facilitating their import into peroxisomes by docking to the peroxisomal membrane where they interact with PEX14. However, under conditions such as nutrient starvation, PEX14 has been reported to interact with LC3, a key component involved in autophagosome formation. This interaction aids in the recognition and degradation of damaged or unnecessary peroxisomes via pexophagy [29,53] (Figure 1B). Optineurin (OPTN) is a multifunctional adaptor involved in various autophagic pathways. Recent studies have suggested that the C-terminal domain of OPTN can bind to PEX14, thereby initiating a cell-type-specific process known as pexophagy. This interaction highlights the role of OPTN as a critical mediator in targeting peroxisomes for degradation under specific cellular conditions. PEX14 serves as an interface linking OPTN to cell-type-selective pexophagy [54]. Further exploration of the molecular mechanisms governing OPTN-mediated pexophagy helps to extend our knowledge of disorders linked to peroxisome dysfunction and OPTN-related abnormalities, which will provide valuable insights into potential therapeutic strategies for treating conditions associated with impaired peroxisomal function (Figure 1B).

### 2.3. Pexophagy Regulated by Different Expression Levels of Peroxisomal Proteins

PEX3 is a peroxisomal membrane protein and a key factor in peroxisome biogenesis that acts in collaboration with PEX16 and PEX19 to facilitate the import of peroxisomal membrane proteins and initiate peroxisome formation from the endoplasmic reticulum (ER) [43]. The overexpression of PEX3 induces pexophagy, which is characterized by the formation of ubiquitinated and clustered peroxisomes regardless of the ubiquitination status of PEX3. However, the specific ubiquitinated substrates during this process remain unidentified, and further investigation is needed to elucidate their precise molecular mechanisms [55] (Figure 1C). PEX16 plays a critical role in peroxisome biogenesis and is involved in the early stages of peroxisomal membrane assembly and growth. Research has indicated that PEX16 plays a role in regulating pexophagy, and that the depletion or dysfunction of PEX16 has been associated with the induction of pexophagy. This suggests that PEX16 participates in maintaining the balance between peroxisome proliferation and degradation via mechanisms that influence pexophagy regulation. Further studies are needed to fully understand the specific mechanisms by which PEX16 affects pexophagy and its implications in cellular homeostasis and diseases [56] (Figure 1C). Catalase, an enzyme located in the peroxisomal matrix, protects cells from oxidative damage by converting hydrogen peroxide to water and oxygen. Catalase deficiency, especially during serum starvation or fasting, can lead to elevated levels of ROS, which in turn activate pexophagy [6,57]. This process underscores the vital role of catalase in protecting cells from oxidative damage and maintaining peroxisome homeostasis (Figure 1C). Moreover, the peroxisomal L-bifunctional enzyme (EHHADH) has recently been identified as a regulator of pexophagy. EHHADH deficiency has been shown to induce pexophagy through increased ROS levels [58] (Figure 1C).

### 2.4. PMP70 Ubiquitination-Mediated Pexophagy

The 70-kDa peroxisomal membrane protein (PMP70), also known as ATP-binding cassette subfamily D member 3 (ABCD3), plays a pivotal role in peroxisome function by transporting crucial substrates across the peroxisomal membrane. The protein is essential for various metabolic processes such as fatty acid metabolism, bile acid synthesis, and the detoxification of xenobiotics. These functions are important for maintaining cellular balance and promoting the overall health of organisms [59,60,61]. Several studies have demonstrated that PMP70 is ubiquitinated in response to cellular stress or pharmacological treatment. Ubiquitinated PMP70 is recognized by autophagy receptors such as NBR1 and p62, which facilitate the targeting of peroxisomes for degradation through pexophagy [27,36]. Under amino acid starvation, PMP70 is a substrate of the ubiquitin ligase, PEX2, which leads to ubiquitination and triggers pexophagy [27]. In response to mTOR inhibition, the ubiquitin ligase MARCH5 binds to PEX19, targeting it to the peroxisome where it ubiquitinates PMP70 and inducing pexophagy [36] (Figure 1D).

## 3. Oxidative Stress and Pexophagy

**Figure 2:**
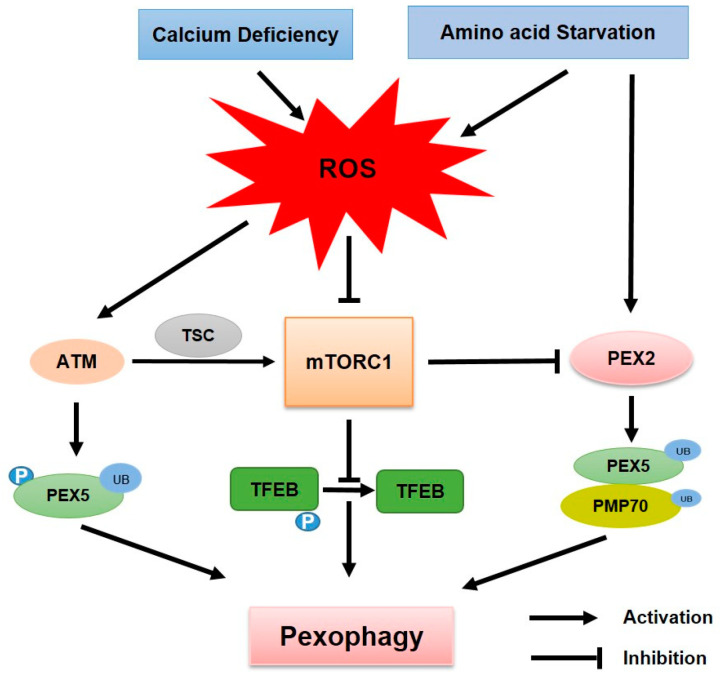
**ROS function as signaling mediators in pexophagy.** ROS induce pexophagy through the mTORC1 signaling pathway under oxidative stress conditions due to calcium deficiency and amino acid starvation. ROS activate ATM, which phosphorylates and ubiquitinates PEX5, leading to its degradation via pexophagy. ATM suppresses mTORC1 activity via the TSC complex. Simultaneously, ROS suppress mTORC1 activity, inducing the dephosphorylation of the transcription factor EB (TFEB), thereby promoting its nuclear translocation and transcriptional activation of autophagy-related genes, leading to pexophagy. Additionally, mTORC1 inhibition influences the ubiquitination of peroxisomal proteins, such as PMP70 and PEX5, which is mediated by PEX2, further promoting pexophagy.

**Figure 3:**
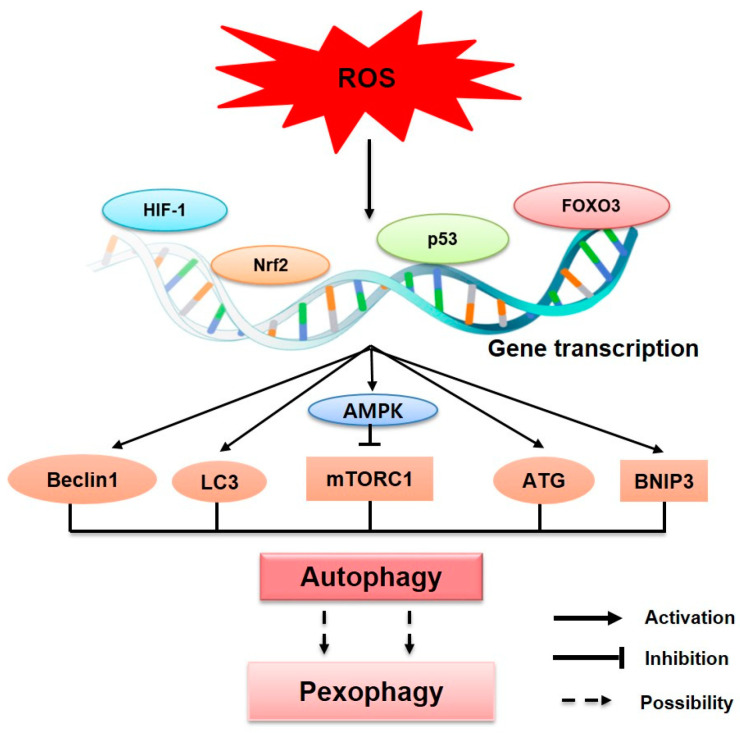
**ROS regulate the transcription of genes involved in autophagy.** ROS trigger various transcription factors, including HIF-1, Nrf2, p53, and FOXO3, to modulate gene transcription. These factors translocate to the nucleus and interact with different signaling pathways, including autophagy-related pathways such as the AMPK and mTORC1 signaling pathways. Autophagy-related proteins such as Beclin1, LC3, ATG proteins, and BNIP3 are regulated through these pathways in response to oxidative stress, which might regulate the selective autophagic degradation of peroxisomes, known as pexophagy.

An imbalance between ROS generation and antioxidant capacity leads to oxidative stress, resulting in excessive ROS levels. Chronic oxidative stress accelerates the aging process and leads to the development of several diseases, including diabetes, cancer, heart disease, and neurological disorders [62]. Peroxisomes play a vital role in generating and scavenging intracellular ROS. They produce ROS as byproducts of fatty acid oxidation (FAO) and contain detoxifying enzymes, such as catalase. ROS cause structural and functional abnormalities in peroxisomes by damaging many of the proteins and lipids in the peroxisomal membrane [63]. Oxidative damage is exacerbated when peroxisomes lose their function because they are unable to detoxify ROS. In recent years, several studies have suggested that ROS accumulation stimulates pexophagy [7,48]. Under oxidative stress, the N-terminal cysteine of ACAD10 is translocated to damaged peroxisomes, where it binds to p62 and subsequently recruits GABARAP or LC3 to autophagic membranes, inducing pexophagy [64]. This section summarizes the pexophagy pathways associated with oxidative stress, with a particular emphasis on the role of ROS (Figure 2 and Figure 3).

### 3.1. ROS Originating from Various Cellular Compartments

Numerous biological components generate ROS from the ER, mitochondria, and peroxisomes known as the three major redox-sensitive organelles [65]. These organelles produce ROS that can be released via aquaporins or unknown protein channels [66]. Although ROS from peroxisomes and mitochondria have been well studied as contributors to pexophagy induction under stress conditions, the precise role of ER-derived ROS in pexophagy remains unclear. Mitochondrial ROS are generated in the electron transport chain during oxidative phosphorylation and act as signaling molecules. Studies have shown that the depletion of the mitochondrial protein HSPA9 in SH-SY5Y cells increases ROS levels [67], and the loss of HSPA9 in these cells induces pexophagy due to peroxisomal ROS accumulation [39]. This suggests that ROS diffuse from the mitochondria to the peroxisomes and promote pexophagy. In addition, elevated levels of ROS within the peroxisomal lumen can activate Stub1-mediated pexophagy [68]. Given the dynamic ROS transfer among mitochondria, peroxisomes, and the ER, ER-derived ROS may play a role in the regulation of pexophagy. Further studies are needed to clarify whether ER-derived ROS directly or indirectly influence pexophagy pathways.

### 3.2. ROS Enhance Pexophagy Through Various Signaling Pathways

ROS serve as critical triggers for the initiation of pexophagy by signaling through different pathways. Upon activation by ROS, ataxia–telangiectasia mutated (ATM) translocates to damaged peroxisomes, where it represses mTORC1 and facilitates the mono-ubiquitination of PEX5 [48]. Consequently, ubiquitinated PEX5 is recognized by pexophagy receptors, such as NBR1 and p62, to induce pexophagy [48] (Figure 2). During amino acid starvation, oxidative stress is induced as ROS levels markedly increase [27,69]. Under amino acid starvation conditions, PEX2 is upregulated and functions as an E3 ubiquitin ligase that ubiquitinates PEX5 and PMP70. Ubiquitinated proteins are subsequently recognized by NBR1 during pexophagy [27] (Figure 2). Calcium deficiency in the medium decreases the catalytic activity of peroxisomal catalase, resulting in the accumulation of ROS, which inhibits mTOR activity and activates the transcription factor EB (TFEB). The inactivation of mTORC1 inhibits TFEB phosphorylation and promotes its nuclear translocation into the nucleus. TFEB stimulates the transcription of genes involved in lysosomal biogenesis and autophagy, enhancing autophagic flux, which leads to pexophagy to remove the oxidative stress present within the cell [7] (Figure 2). Interestingly, in response to peroxisomal ROS, ATM activates the tuberous sclerosis complex (TSC), which localizes to peroxisomes, inhibits mTORC1, and promotes autophagy [70]. Given that the mTORC1 pathway has been implicated in the regulation of pexophagy by controlling PEX2 expression, these findings suggest that mTORC1 serves as a central regulator of pexophagy by coordinating cellular responses to oxidative stress and nutrient deprivation. Although these interactions highlight an important link between ROS, mTORC1 activity, and pexophagy, further studies are required to determine the precise mechanisms by which ROS regulate pexophagy.

### 3.3. ROS Induce Autophagy by Regulating Gene Expression

ROS trigger the activation of transcription factors such as hypoxia-inducible factor (HIF-1α), nuclear factor erythroid 2-related factor 2 (Nrf2), p53, and forkhead box O-3 (FOXO3) [71,72,73,74]. These transcription factors induce the expression of genes required for autophagy induction, including BECN1, LC3, and SQSTM1 [75,76,77] (Figure 3). HIF-1α is rapidly degraded by the oxygen-dependent prolyl hydrolases (PHDs), through ubiquitin–proteasome degradation in normal oxygen levels. However, this degradation is inhibited under hypoxic conditions, and HIF-1α is stabilized and accumulates [78]. The stabilized HIF-1α translocates to the nucleus, where it activates genes that support survival under hypoxia, such as those involved in angiogenesis and glucose metabolism. Notably, increased ROS levels can suppress PHDs, allowing HIF-1α to accumulate and activate target genes for pro-autophagic proteins such as BNIP3 and NIX [78,79], which are well-known mitophagy/pexophagy receptors that induce selective autophagy [80,81]. Under oxidative stress, Nrf2 is stabilized and translocated to the nucleus to activate the expression of antioxidant and cytoprotective genes to neutralize ROS and restore the cellular redox balance [82]. Additionally, Nrf2 directly regulates mTOR activity, establishing a connection between the oxidative stress response and autophagy [83,84]. p53 plays a dual role in promoting or inhibiting autophagy depending on its subcellular localization. When oxidative stress occurs, DNA damage leads to the activation of p53, which enhances AMPK activity and, in turn, inhibits mTOR signaling. This promotes autophagy as a protective response against oxidative damage [85]. In addition, nuclear p53 induces the transcriptional activation of autophagy-related genes, such as damage-regulated autophagy modulator (DRAM) and TP53-induced glycolysis and apoptosis regulator (TIGAR), further facilitating autophagy [86,87]. Similarly, oxidative stress triggers the nuclear translocation of FOXO3 and induces the transcription of autophagy-related genes such as LC3, BNIP3, and ATG. This activation helps to reduce oxidative stress by removing ROS and preventing further cellular damage [88] (Figure 3).

The upregulation of these autophagy-related genes promotes specific forms of autophagy such as pexophagy. The accumulation of ROS triggers cellular stress responses that activate autophagic pathways, including those that selectively degrade peroxisomes, helping to maintain cellular homeostasis and mitigate oxidative damage. However, the exact mechanism by which ROS regulate pexophagy has not yet been fully elucidated.

## 4. Role of Pexophagy and Oxidative Stress in Neurological Diseases

Both peroxisomes and oxidative status are critical for cellular stability, specifically in an organ with a high demand for energy such as the brain [89,90]. This notion indicates that the regulatory balance of pexophagy and oxidative stress is essential for supporting proper neuronal health, and disruptions in this balance contribute to the progression of neurodegenerative diseases.

Peroxisomes are crucial for the formation of cellular membranes and myelin sheaths in the CNS. In addition, the ether phospholipids produced by peroxisomes are essential for maintaining proper function of glia and neurons [91]. Pexophagy plays a critical role in protecting neurons from oxidative damage by selectively degrading dysfunctional peroxisomes that could otherwise contribute to excessive ROS production [20]. The dysregulation of pexophagy can exacerbate oxidative stress and cellular damage, contributing to disease progression. In addition to their role in lipid synthesis, peroxisomes are central to cellular oxidative metabolism, including the β-oxidation of very-long-chain fatty acids (VLCFAs) and the detoxification of ROS. Impaired pexophagy can lead to the accumulation of dysfunctional peroxisomes, resulting in elevated levels of ROS and VLCFAs, both of which are observed in neurodegenerative diseases such as Alzheimer’s disease and Parkinson’s disease [89,90]. Moreover, the failure to remove excessive or damaged peroxisomes may contribute to mitochondrial dysfunction, another critical factor in neurodegeneration. On the other hand, neurodegenerative diseases are characterized by the accumulation of misfolded proteins such as amyloid-β, tau, and α-synuclein [92]. Recent studies suggest that peroxisomal dysfunction and impaired pexophagy can indirectly affect proteostasis by influencing cellular redox states and lipid metabolism, indicating that the dysregulation of pexophagy may promote protein aggregation and neurotoxicity [90,93]. In addition, as a specialized form of autophagy, pexophagy is also influenced by general autophagy pathways, including those regulated by mTOR signaling. For example, in ALS, mutations in key autophagy regulators, such as TDP-43 and FUS, may also impair selective processes including pexophagy [94]. Similarly, reduced autophagic activity in aging may further contribute to the impaired autophagic degradation of peroxisomes, resulting in the progressive neuronal damage exacerbating neurodegeneration [95].

Several signs of neurodegeneration associated with pexophagy gene knockout include growth retardation, abnormal reflexes, premature death, and progressive motor deficits. These manifestations highlight the critical role of pexophagy genes in maintaining neural health and function [96,97,98]. Pharmacological promotion of pexophagy through the activation of key regulators may be beneficial for the treatment of neurological disorders [20]. In addition, targeting the mTOR signaling pathway may offer a therapeutic angle for neurodegenerative diseases since mTOR inhibition has been shown to induce pexophagy.

Certain proteins that regulate pexophagy have been implicated in neurological disorders, such as USP30, MARCH5, and HSPA9 [99,100,101]. However, there was no direct evidence linking their role in these disorders to their regulation of pexophagy. Despite growing interest in pexophagy and its effects in neurological diseases, its precise role in neurological diseases has not been well studied. The mechanisms underlying the pexophagy crosstalk in neurological pathology require further investigation, for example, identifying specific biomarkers for pexophagy dysregulation in neurodegenerative diseases. A deeper understanding of the molecular mechanisms and therapeutic potential of pexophagy may lead to novel treatments for neurodegenerative diseases and other diseases associated with peroxisomal dysfunction.

Oxidative stress refers to the imbalance between free radicals and antioxidants in the body. As the CNS consumes large amounts of oxygen and generates abundant free radicals, it is particularly sensitive to such imbalances, and they can contribute to the development and progression of neurological diseases. It is well known that oxidative stress induces protein misfolding and aggregation by promoting the oxidative modifications of proteins that hinder their proper folding and clearance. In addition, oxidative stress can impair the function of molecular chaperones and proteasomal activity, further disrupting proteostasis and accelerating neurodegeneration [102]. Mitochondria are both a major source and a target of ROS, and excessive ROS generated by damaged mitochondria can impair neuronal energy metabolism. For example, in Parkinson’s disease, mutations in genes, such as PINK1 and Parkin, disrupt mitochondrial quality control, leading to oxidative damage and neuronal loss [103]. Moreover, lipid peroxidation products, such as malondialdehyde and 4-hydroxynonenal, are toxic messengers that propagate and amplify oxidative damage, which further contribute to neuronal dysfunction [104]. Inflammation and oxidative stress are closely interconnected and play pivotal roles in the pathogenesis of neurological diseases. Chronic neuroinflammation amplifies oxidative stress, and in turn, excessive oxidative stress exacerbates inflammation. This vicious cycle contributes to neuronal dysfunction and degeneration [105]. As oxidative stress is one of common features shared by several numerous neurodegenerative diseases such as Alzheimer’s disease, Parkinson’s disease, and amyotrophic lateral sclerosis [89], numerous preclinical and clinical investigations are exploring antioxidant therapeutic strategies. However, the effectiveness of antioxidant therapy for these conditions remains a topic of debate, largely due to inconclusive results from clinical trials.

Despite the growing recognition of the interplay between pexophagy and oxidative stress in neurological diseases, many aspects remain unexplored. Recently, TFEB activation was shown to be a promising therapeutic strategy for Alzheimer’s disease. Pexophagy occurs through TFEB activation for functional adaptation to oxidative stress [7,106].

Like mitotherapy, which is a promising approach for preventing neurodegeneration [107], pexotherapy may constitute a new therapeutic axis to address aging but also to prevent and/or treat age-related diseases [108]. For example, 7-ketocholesterol (7KC) and 7β-hydroxycholesterol (7β-OHC) are toxic oxysterols produced by cholesterol autoxidation, which has been implicated in neurological disorders such as Alzheimer’s disease [109,110]. These two oxysterols can induce oxidative stress and alter the quality, quantity, and function of peroxisomes [110,111,112]. However, treatment with certain molecules can prevent peroxisomal damages [108,110]. Although there is currently a lack of definitive evidence for peroxisome-targeted therapies, these findings provide a direction for future peroxisome-targeted therapies and may expand the exploration of pexophagy and oxidative stress, providing potential strategies for addressing neurological diseases.

## 5. Conclusions

In this review, we summarize the current state of the research on the regulatory mechanisms between pexophagy and the role of oxidative stress, particularly ROS. Although significant progress has been achieved in understanding the interplay between pexophagy and oxidative stress, it is far from being completely elucidated, and their relationship remains a critical but underexplored area in neurological disease research. Several questions still remain, including how oxidative stress signals are integrated into pexophagy pathways and the disease-specific dynamics of these interactions. Addressing these challenges requires innovative approaches, including the identification of specific biomarkers for peroxisomal health and advanced methods for distinguishing pexophagy from general autophagy. Further studies are needed to explore the precise mechanisms linking pexophagy and oxidative stress, which may aid in the development of novel therapeutic strategies aimed at restoring cellular homeostasis, reducing oxidative damage, and protecting neuronal function. Moreover, clinical interventions targeting these pathways would provide more effective and promising outcomes in the treatment of neurodegenerative diseases.
